# Supplementary material for: Insect population dynamics under Wolbachia-induced cytoplasmic incompatibility: Puzzle more than buzz in Drosophila suzukii
Source: PLoS One. 2024 Mar 12;19(3):e0300248. doi: 10.1371/journal.pone.0300248 (PMC10931435; doi:10.1371/journal.pone.0300248)
Supplement: S1 Fig — Black square represents area boundaries. Green and red arrows indicate which flies are counted and which are not. Area 1: flies appearing on the front and on the rear panels, Area 2: individuals appearing on the whole surface except the left side and the bottom of the cage and Area 3: only individuals appearing on the front panel. (DOCX) [file pone.0300248.s001.docx]

**S1 Fig**

Area 1 Area 2


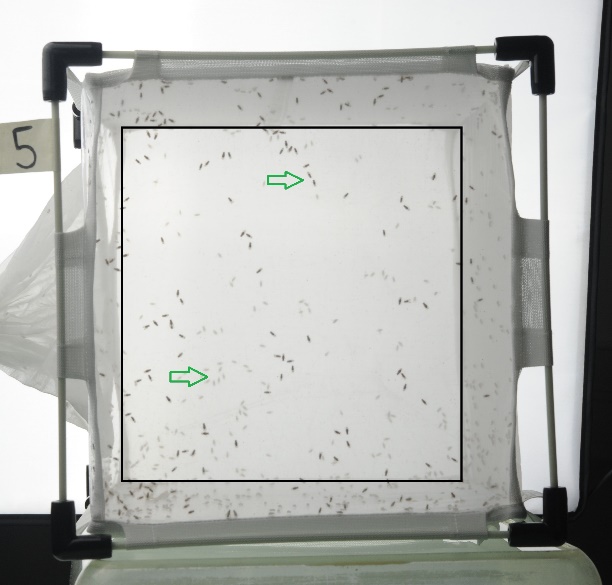

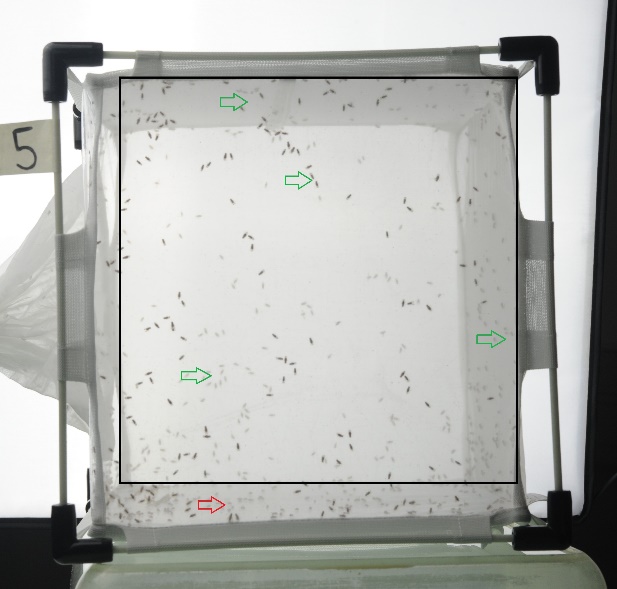


Area 3


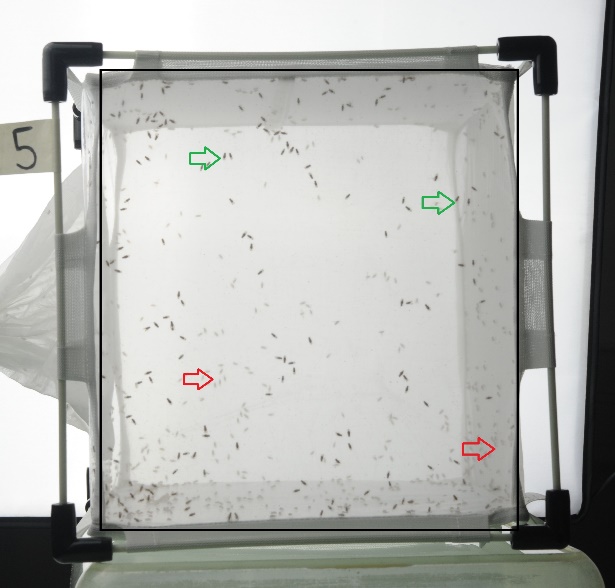


**Different areas used for the census method.** Black square represents area boundaries. Green and red arrows indicate which flies are counted and which are not. Area 1 : flies appearing both on the front and on the rear panels, Area 2 : individuals appearing on the whole surface except the left side and the bottom of the cage and Area 3 : only individuals appearing on the front panel.
