# Supplementary material for: Insect population dynamics under Wolbachia-induced cytoplasmic incompatibility: Puzzle more than buzz in Drosophila suzukii
Source: PLoS One. 2024 Mar 12;19(3):e0300248. doi: 10.1371/journal.pone.0300248 (PMC10931435; doi:10.1371/journal.pone.0300248)
Supplement: S2 Fig — Here, are plotted the negative first derivative of the fluorescence (-d(RFU)/dt (10 3) versus temperature, peaks corresponding to the melting temperature Tm of Wolbachia DNA (Blue line: wSuz, green line: wTei, red line: wHa present in third transinfected line of Drosophila suzukii, which was not used for these experiments). FRU means relative fluorescence units. (DOCX) [file pone.0300248.s002.docx]

**S2 Fig**

**
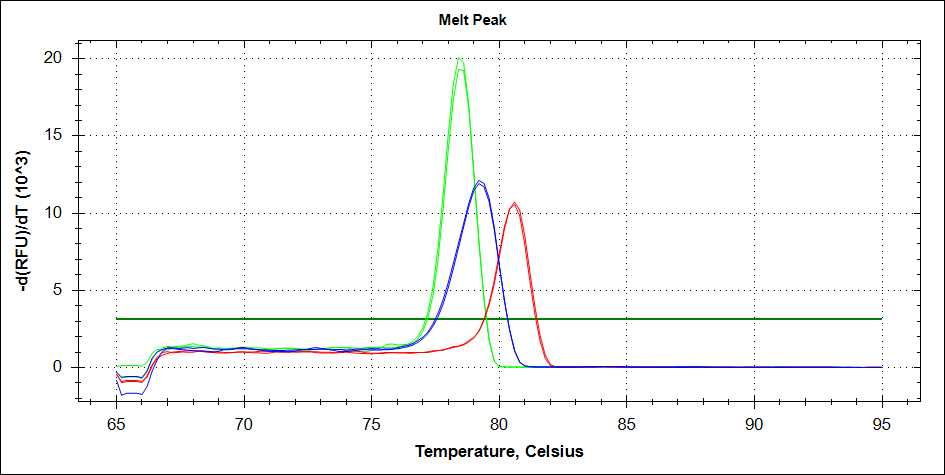
**

**Melting curves analysis of DNA fragments after PCR for different *Wolbachia* strains in *Drosophila suzukii*.**

Here, are plotted the negative first derivative of the fluorescence (-d(RFU)/dt (10 ^3^) *versus* temperature, peaks corresponding to the melting temperature Tm of *Wolbachia* DNA (Blue line: *w*Suz, green line: *w*Tei, red line: *w*Ha present in third transinfected line of *Drosophila suzukii*, which was not used for these experiments). FRU means relative fluorescence units.
